# Perioperative Chemotherapy for Gastro-Esophageal or Gastric Cancer: Anthracyclin Triplets versus FLOT

**DOI:** 10.3390/cancers16071291

**Published:** 2024-03-26

**Authors:** Julie F. M. Geerts, Charlène J. van der Zijden, Pieter C. van der Sluis, Manon C. W. Spaander, Grard A. P. Nieuwenhuijzen, Camiel Rosman, Hanneke W. M. van Laarhoven, Rob H. A. Verhoeven, Bas P. L. Wijnhoven, Sjoerd M. Lagarde, Bianca Mostert

**Affiliations:** 1 Department of Surgery, Catharina Hospital, 5623 EJ Eindhoven, The Netherlands; julie.geerts@catharinaziekenhuis.nl (J.F.M.G.); grard.nieuwenhuijzen@catharinaziekenhuis.nl (G.A.P.N.); 2 Department of Surgery, Erasmus MC Cancer Institute, 3015 GD Rotterdam, The Netherlands; c.vanderzijden@erasmusmc.nl (C.J.v.d.Z.); b.wijnhoven@erasmusmc.nl (B.P.L.W.); s.lagarde@erasmusmc.nl (S.M.L.); 3 Department of Gastroenterology and Hepatology, Erasmus University Medical Center, 3015 GD Rotterdam, The Netherlands; v.spaander@erasmusmc.nl; 4 Department of Surgery, Radboud University Medical Center, 6525 GA Nijmegen, The Netherlands; camiel.rosman@radboudumc.nl; 5 Department of Medical Oncology, Amsterdam UMC Location University of Amsterdam, 1081 HV Amsterdam, The Netherlands; h.vanlaarhoven@amsterdamumc.nl (H.W.M.v.L.); r.verhoeven@iknl.nl (R.H.A.V.); 6 Cancer Center Amsterdam, Cancer Treatment and Quality of Life, 1105 AZ Amsterdam, The Netherlands; 7 Department of Research & Development, Netherlands Comprehensive Cancer Organization (IKNL), 3511 LC Utrecht, The Netherlands; 8 Department of Medical Oncology, Erasmus MC Cancer Institute, 3015 GD Rotterdam, The Netherlands; b.mostert@erasmusmc.nl

**Keywords:** esophageal cancer, gastro-esophageal cancer, gastric cancer, neoadjuvant chemotherapy, esophagectomy

## Abstract

**Simple Summary:**

In the current study, we have collected real-world population data from the Netherlands Cancer Registry of patients who underwent perioperative anthracyclin triplets or FLOT. Our study showed no significant overall survival improvement for FLOT-treated patients compared to anthracyclin triplets, despite more staging laparoscopies in the first group. However, FLOT patients demonstrated higher rates of neoadjuvant therapy completion, proceeding to adjuvant therapy, and increased pathological complete response rates. Even though survival difference failed to reach statistical significance, we believe that our findings hold significance as they mirror the outcomes observed in clinical practice, outside the controlled environment of a clinical trial.

**Abstract:**

*Background:* The FLOT4-AIO trial (2019) showed improved survival with perioperative fluorouracil, leucovorin, oxaliplatin, and docetaxel (FLOT) compared to anthracyclin triplets in gastric cancer treatment. It is unclear whether these results extend to real-world scenarios in the Netherlands. This study aimed to compare outcomes of perioperative FLOT to anthracyclin triplets in a real-world Dutch gastric cancer population. *Methods:* Patients diagnosed with resectable (cT2-4a/cTxN0-3/NxM0) gastric or gastro-esophageal junction carcinoma between 2015–2021 who received neoadjuvant FLOT or anthracyclin triplets were selected from the Netherlands Cancer Registry. The primary outcome was overall survival (OS), analyzed through multivariable Cox regression. Secondary outcomes included pathological complete response (pCR), neoadjuvant chemotherapy cycle completion, surgical resection rates, and adjuvant therapy. *Results:* Adjusted OS showed no significant survival benefit (HR = 0.88, 95% CI 0.77–1.01, *p* = 0.07), even though the median OS was numerically improved by 8 months with FLOT compared to anthracyclin triplets (48.1 vs. 39.9 months, *p* = 0.16). FLOT patients were more likely to undergo diagnostic staging laparoscopies (74.2% vs. 44.1%, *p* < 0.001), had higher rates of completing neoadjuvant chemotherapy (OR = 1.35, 95% CI 1.09–1.68, *p* = 0.007), receiving adjuvant therapy (OR = 1.34, 95% CI 1.08–1.66, *p* = 0.08), and achieving pCR (OR = 1.52, 95% CI 1.05–2.20, *p* = 0.03). No significant differences were observed in (radical) resection rates. *Conclusion(s):* Real-world data showed no significant OS improvement for FLOT-treated patients compared to anthracyclin triplets, despite more staging laparoscopies. However, FLOT patients demonstrated higher rates of neoadjuvant therapy completion, proceeding to adjuvant therapy, and increased pCR rates. Therefore, we recommend the continued use of neoadjuvant FLOT therapy in the current clinical setting.

## 1. Introduction

Treatment and survival of patients with gastric or gastro-esophageal junctional (GEJ) carcinoma have shown progress over the last decades. The introduction of perioperative chemotherapy for resectable disease and centralization of gastric surgery have improved patients’ prognosis as shown in clinical trials [1,2]. Despite these improvements, 5-year survival rate for potentially curable gastric cancer patients in the Netherlands has only seen a minor increase from 27% during 2000–2010 to 34% in 2011–2020 [3]. Thus, the prognosis for these patients remains poor, and a substantial number are exposed to the toxicity of chemotherapy without any benefits [4,5].

In 2006, the MAGIC trial investigated the effect of perioperative chemotherapy (epirubicin 50 mg/m^2^, cisplatin 60 mg/m^2^, and fluorouracil 200 mg/m^2^) on survival of patients with locally advanced resectable gastric or GEJ carcinoma [6]. This study showed an improved 5-year survival following perioperative chemotherapy (36%) compared to surgery alone (23%). Subsequent research demonstrated equivalent survival when fluorouracil was replaced by oral capecitabine (1000 mg/m^2^) [7,8]. Oxaliplatin was also found to be similarly effective as cisplatin in the advanced setting [9]. Consequently, various perioperative chemotherapy regimens were employed, encompassing combinations of epirubicin, cisplatin, or oxaliplatin, plus fluorouracil or capecitabine (ECF/ECX/EOX). These anthracycline-based triplets were considered the standard of care between 2006–2019 in Europe for patients with locally advanced gastric or GEJ adenocarcinoma [10].

Subsequently, the FLOT4-AIO trial was conducted, in which patients were randomized between perioperative ECF/ECX or FLOT (fluorouracil, leucovorin, oxaliplatin, and docetaxel) followed by surgical resection [11]. This study demonstrated a significantly improved overall survival (OS) in favor of FLOT (35 vs. 50 months). In addition, higher pathological complete response (pCR) rates were seen after treatment with FLOT [11,12]. After an implementation period from 2017–2019, FLOT is now standard treatment for this patient population in the Netherlands and Europe [13,14].

However, it remains unclear whether the results of the FLOT4-AIO trial translate to similar improvements and toxicity rates in a “real-life” gastric cancer population. Therefore, the aim of this study is to compare the outcomes of patients treated with anthracyclin triplets to those with FLOT on a population-based level.

## 2. Materials and Methods

### 2.1. Study Design and Patients

The Netherlands Cancer Registry (NCR) is a nationwide population-based cancer registry that covers the entire Dutch population of more than 17 million people. The NCR is based on notifications of all newly diagnosed malignancies in the Netherlands, and trained NCR employees routinely extract information on diagnosis, tumor stage, and treatment from medical records. Patients diagnosed with resectable (cT2-4a/cTx, cN0-3/Nx, cM0) gastric, distal esophageal, or GEJ cancer between 2015–2021 were selected from the NCR.

### 2.2. Perioperative Chemotherapy

Patients were included if they started at least one cycle of neoadjuvant anthracyclin triplets or FLOT. Some patients were treated with neoadjuvant chemotherapy without surgical intervention, while others proceeded to surgical resection followed by adjuvant chemotherapy. Resection was omitted in the case of disease progression, the detection of irresectable or metastatic disease after neoadjuvant chemotherapy or during explorative surgery, or when patients were deemed unfit for surgery. Treatment with anthracyclin triplets included: epirubicin, capecitabin, and oxaliplatin (EOX); epirubicin, cisplatin, and 5-fluorouracil (ECF); epirubicin, capecitabin, and cisplatin (ECC); or epirubicin, oxaliplatin, and 5-fluorouracil (EOF).

### 2.3. Pathological Assessment

Tumor staging was performed according to the UICC 7th and 8th TNM staging manual [15,16]. Radical resection (R0) was defined as no contact between tumor and surgical margin (clearance of ≥1 mm), and a microscopically irradical resection (R1) was defined as <1 mm contact between tumor and surgical margin [17]. A macroscopically irradical resection (R2) was defined as visible residual tumor which was left behind during surgery and could not be resected because of ingrowth in surrounding organs or tissues.

### 2.4. Outcomes

The primary outcome was OS of patients treated with anthracyclin triplets or FLOT chemotherapy. Survival differences were also calculated for the subset of patients who met the major inclusion criteria of the FLOT4-AIO trial (>cT1 and/or cN+ tumor not invading adjacent structures or organs, ECOG score ≤ 2, absence of peritoneal metastatic disease during diagnostic laparoscopy (DLS), no history of secondary malignant diseases or heart failure, and complete data on comorbidities and vital status). Secondary outcomes included the proportion of patients that completed the full neoadjuvant chemotherapy regimen, defined as 100% of scheduled neoadjuvant cycles, allowing for dose reductions, and delays of chemotherapy cycles or surgical resection, defined as an interval of ≥17 weeks (anthracyclin triplets) or ≥16 weeks (FLOT) between the last chemotherapy and resection. Other secondary endpoints were the proportion of patients with pCR (defined as ypT0N0), the proportion of patients undergoing surgical resection, rates of radical resection, 30- and 90-day mortality, the proportion of patients receiving adjuvant therapy, and those who underwent primary resection within the study period. To assess whether fewer patients with poor prognostic factors were offered primary resection in the FLOT era, we compared the baseline characteristics of neoadjuvantly treated patients with patients undergoing primary resection in both eras.

### 2.5. Statistical Analyses

Patient and tumor characteristics were analyzed using descriptive statistics and were presented as mean, median with interquartile range (IQR), or frequencies (%). Differences in patient characteristics were analyzed using Chi-squared or Fisher exact tests when appropriate. Survival was reported in months and was calculated from start of neoadjuvant chemotherapy until date of death or last day of follow-up, using the Kaplan–Meier method and multivariable Cox regression analysis. Median follow-up was calculated from date of diagnosis. Univariable and multivariable logistic regression analyses were used to investigate pCR, completion of neoadjuvant treatment, proportion of patients proceeding to resection and adjuvant treatment, radical resections, and 30-/90-day mortality. All variables with a *p*-value < 0.05 in the univariable analysis were included in multivariable regression analyses. Survival data were expressed as hazard ratios (HRs) with 95% confidence intervals (CI), while secondary outcomes were expressed as odds ratios (ORs) with 95% CI. Statistical significance was defined as a *p*-value < 0.05. Statistical analysis was performed with the use of R version 4.0.0 (R: A language and environment for statistical computing. R Foundation for Statistical Computing, Vienna, Austria).

## 3. Results

**Figure 1:**
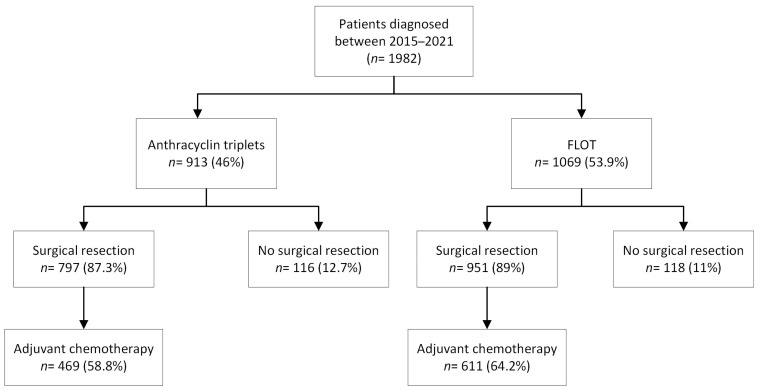
Flowchart of patients treated with neoadjuvant anthracyclin triplets or FLOT chemotherapy. FLOT: fluorouracil, leucovorin, oxaliplatin, and docetaxel.

A total of 1982 patients were included in this study. Baseline characteristics are presented in Table 1, and patient and tumor characteristics from the current study and the FLOT4-AIO trial are shown in Table S1. Treatment consisted of anthracyclin triplets in 913 patients (46.0%) and FLOT in 1069 patients (53.9%) (Figure 1). Patients treated with anthracyclin triplets underwent fewer diagnostic laparoscopies than patients treated with FLOT (44.1% vs. 74.2%, *p* < 0.001).

### 3.1. Survival and Prognostic Factors

**Figure 2:**
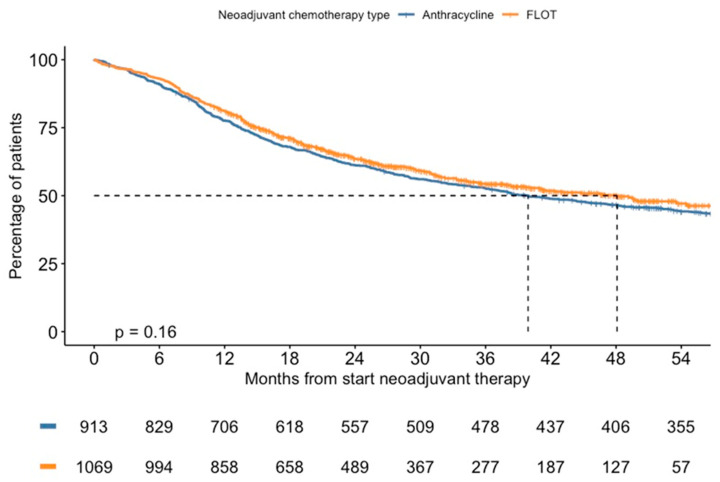
Overall survival over time for patients treated with neoadjuvant anthracyclin triplets or FLOT chemotherapy.

Median OS for patients treated with anthracyclin triplets was 39.9 months (95% CI 33.3–46.8) and 48.1 months (95% CI 38.4 not reached) for FLOT-treated patients (*p* = 0.16), with 3-year survival rates of 52.8% and 54.4%, respectively (Figure 2). The median follow-up for patients treated with anthracyclin triplets was 39 months (IQR 14–72) and 23 months (IQR 15–38) for FLOT.

Univariate analysis showed that increasing age (HR = 1.01, 95% CI 1.01–1.02, *p* = 0.0001) and WHO performance status of ≥2 (HR = 1.71, 95% CI 1.27–2.30, *p* = 0.0004) were significantly associated with worse OS. Likewise, clinical T3 (HR = 1.23, 95% CI 1.07–1.43, *p* = 0.005), T4A (HR = 1.44, 95% CI 1.11–1.86, *p* = 0.006), clinical N+ (HR = 1.36, 95% CI 1.20–1.54, *p* < 0.0001), Lauren classification diffuse type (HR = 1.72, 95% CI 1.50–1.98, *p* < 0.0001), unknown histology (HR = 1.35, 95% CI 1.11–1.64, *p* = 0.003), and differentiation grade ≥3 (HR = 1.61, 95% CI 1.38–1.88, *p* < 0.0001) were significantly associated with decreased OS. In multivariable analysis, these factors remained significant (Table 2). Undergoing a diagnostic staging laparoscopy was univariably significantly associated with worse OS (HR = 1.14, 95% CI 1.00–1.30, *p* = 0.04). However, this did not remain a significant factor in multivariable analysis. Sex, number of comorbidities, tumor location, and tumor morphology were not significantly associated with OS.

Adjusted for the aforementioned factors (age, WHO performance status, clinical T- and N-stage, Lauren classification, differentiation grade, and DLS), multivariable analysis showed no OS difference between both groups (HR = 0.88, 95% CI 0.77–1.01, *p* = 0.07).

A subgroup analysis was performed on 1155 patients meeting the main inclusion and exclusion criteria of the FLOT4-AIO trial. Within this subgroup, 463 patients (40.1%) were treated with anthracyclin triplets and 692 (59.9%) with FLOT. Again, no statistically significant difference in median OS could be demonstrated between anthracyclin triplets (41 months) and FLOT (49.5 months) (*p* = 0.17). Multivariable Cox regression analysis adjusted for clinical T-stage, clinical N-stage, Lauren classification, differentiation grade, age, performance status, and DLS, confirmed no significant survival benefit associated with treatment using FLOT (HR = 0.89, 95% CI 0.71–1.03, *p* = 0.10).

### 3.2. Neoadjuvant Chemotherapy Cycles

The proportion of patients that completed the full neoadjuvant chemotherapy regimen was significantly higher with FLOT (77.6% vs. 73.1%, *p* = 0.02). In multivariable logistic regression analysis adjusted for age, performance status, number of comorbidities, clinical T-stage, clinical N-stage, tumor location, Lauren classification, differentiation grade, and undergoing DLS, this effect remained statistically significant (OR = 1.32, 95% CI 1.05–1.66, *p* = 0.02). Delay of any chemotherapy cycle or delay to surgical resection could only be calculated for patients who underwent resection and occurred in 12.9% of patients treated with anthracyclin triplets and 14.2% of patients treated with FLOT (*p* = 0.95).

### 3.3. Secondary Outcomes

In total, 1748 patients (88.2%) underwent surgical resection after neoadjuvant chemotherapy; 87.3% of patients after anthracyclin triplets; and 89.0% after FLOT (*p* = 0.26) (Table 3). Multivariable logistic regression adjusted for age, performance status, number of comorbidities, clinical T-stage, clinical N-stage, tumor location, Lauren classification, differentiation grade, and undergoing DLS showed a similar outcome (OR = 1.34, 95% CI 0.98–1.82, *p* = 0.07). No significant differences were seen in radical resection rates.

Significantly more patients had a pCR after FLOT (9.8% vs. 7.8%, OR = 1.58, 95% CI 1.08–2.32, *p* = 0.02), which corresponds to the significantly higher proportions of ypT0-1 and ypN0 seen after FLOT (*p* < 0.0001 and *p* = 0.0003, respectively). After surgical resection, the proportion of patients that underwent adjuvant therapy was significantly higher in the FLOT group (OR = 1.37, 95% CI 1.10–1.71, *p* = 0.005).

### 3.4. Primary Surgical Resection

During the study period, a total of 1053 patients of the entire population underwent primary resection (Tables S2 and S3). The proportion of patients that received primary resection decreased from 39.0% in 2015 to 29.2% in 2021.

## 4. Discussion

This retrospective real-world population study showed no statistical significant improvement in median OS for patients treated with FLOT compared to anthracyclin triplets. Even when examining the subset of patients that closely met the major inclusion and exclusion criteria of the FLOT4-AIO trial, no significant survival difference was observed.

The median OS after FLOT in this study (48.1 months) aligned with the results reported in the FLOT4-AIO trial (50 months). However, median OS after anthracyclin triplets was considerably longer in this study (39.9 months) compared to both the FLOT4-AIO trial (35 months) and the MAGIC trial (25 months). This discrepancy could be influenced by various factors, including differences in perioperative care and surgical outcomes. Notably, when comparing the Dutch patients in the anthracyclin group to the ECF/ECX group of the FLOT4-AIO trial, a higher percentage achieved radical resection in this study (87.6% vs. 78%). Contrary to the trial, this study found no significant difference in radical resection rates between both groups. In addition, a higher percentage of patients in the anthracyclin group in the current study received adjuvant treatment compared to the FLOT4-AIO trial (58.9% vs. 52%), and the 90-day mortality in this cohort was also lower for anthracyclin-treated patients (4.0%) than in the trial population (8.0%). These factors may have positively influenced the OS of the anthracycline group, potentially reducing the additional beneficial effect of FLOT over anthracyclin triplets.

The lack of significant OS difference could also be explained by a less stringent selection of patients in regards to WHO performance status or comorbidities for perioperative chemotherapy throughout the years. This trend is supported by the decreasing number of primary resections observed in recent years, indicating a growing preference for perioperative chemotherapy (Table S3). Although significant differences in most baseline characteristics were seen between patients treated with perioperative chemotherapy and primary resection, specific details per treatment arm and year were missing, preventing definitive conclusions (Table S2).

Additionally, adherence to trial treatment protocols may not accurately reflect real-world practice [18]. Neoadjuvant treatment could be less dose-intensive in a real-world setting, potentially due to frailty of treated patients, which cannot be captured in WHO performance status. For instance, in the FLOT4-AIO trial, 19.0% of patients required dose reductions during neoadjuvant treatment. While reliable data on dose reductions for the entire study period were not available in this study, the RealFLOT study reported that 39.8% of patients needed dose reductions, discontinuation, or substitution with a less intensive neoadjuvant chemotherapy regimen [19]. Given the toxicity of FLOT, and potentially more frail patients being considered as candidates for neoadjuvant treatment in the FLOT era, this difference in dose intensity between trial and real-world could be more pronounced in the FLOT-treated patients.

The rate of DLS prior to perioperative chemotherapy was significantly higher in the FLOT group, consistent with the increased implementation of DLS as a method to detect occult peritoneal metastases. Studies have reported that in over 25% of patients undergoing DLS, occult metastases are found [20,21,22,23], leading to changes in treatment plans from curative to palliative approaches [22,23,24,25]. Surprisingly, despite the likelihood that the anthracyclin group may have included more patients with occult metastases due to the lower rate of DLS, survival in this group was not significantly worse than in the FLOT group. Furthermore, whether DLS was performed or not was not a prognostic factor for survival in the multivariate analysis.

A large multicenter study showed that docetaxel increased OS only in the intestinal type cancers, but not in the diffuse type, which is possibly caused by diminished drug-induced microtubule stabilization [26,27]. This study found that 39.8% of patients in the anthracycline group and 35.6% in the FLOT group were diagnosed with diffuse type gastric cancer, while in the FLOT4-AIO trial 27.0% of patients were diagnosed with diffuse type in both treatment arms. The difference in histological subtypes in these studies could have affected the survival outcomes. Since more patients were diagnosed with diffuse type in this study compared to the FLOT4-AIO trial, OS found in the trial could be higher than in this real-world population. In addition to Lauren histological subtype, studies have shown that other molecular subtypes could affect response to chemotherapy [28,29]. Unfortunately, no data on these subtypes were available in the database used for this study.

In the multivariable analysis, a significant difference in pCR rate was found. However, when compared to results of the FLOT4-AIO trial, the pCR rate after neoadjuvant treatment with FLOT was disappointingly low (9.8%). In contrast, the trial reported a pCR rate of 16.0% [11,12]. A small observational study supported these findings, reporting a pCR of 17.4%, based on only eight patients [30]. Another small study found a pCR rate of 20.0%, but only after application of prolonged neoadjuvant chemotherapy [31]. On the other hand, other small real-world studies found more comparable pCR rates to the current study (5.0–14.0%) [32,33,34,35]. This, again, supports the hypothesis that patients selected for trials differ from a real-world population.

To our knowledge, this is the largest study describing the efficacy of FLOT and anthracyclin triplets on a population-based level. Two major strengths of this study include its large cohort and the use of unselected real-world population data. This provides a representative assessment of daily clinical practice in a Western population. Another strength is that in the Netherlands, surgical care for gastric cancer has been centralized since the study period (2013), and quality assurance is ensured through systematic registration and feedback mechanisms concerning procedure volumes and patient outcomes [36,37]. A limitation of this study is the retrospective design, which may introduce inherent biases and limitations associated with retrospective data analysis. Additionally, for certain variables, such as Lauren classification or performance status, more than 10% of data was missing or unknown. This was seen more often in the anthracyclin-treated group, which might have influenced the study outcomes. However, this does reflect the real-world since it possibly shows improved diagnostics or registration practices over time.

## 5. Conclusions

In conclusion, the previously reported significant survival benefit of FLOT over anthracyclin-based perioperative chemotherapy could not be reproduced in this real-world population, despite the large patient cohort and improved diagnostics (DLS) to rule out occult metastases in the FLOT group. This raises concerns over the external validity of the FLOT4-AIO results, and emphasizes the need to carefully select patients for whom completion of the full perioperative chemotherapy regimen seems attainable. Further research should be focused on identifying factors that can predict response to FLOT chemotherapy, as the toxicity and pressure on health care capacity involved in this regimen can only be accepted when significant survival benefit follows.

## Figures and Tables

**Table 1:** Patient and tumor characteristics.

Characteristics	All Patients (*n* = 1982, %)	Anthracyclin Triplets (*n*= 913, %)	FLOT	*p*-Value
			(*n* = 1069, %)	
**Sex**				
Male	1330 (67.1)	597 (65.4)	733 (68.6)	0.13 ^a^
**Age** (median [IQR])	67 (59–73)	66 (59–72)	67 (59–73)	0.02 ^b^
**Tumor type**				
Adenocarcinoma	1665 (84.0)	753 (82.5)	912 (85.3)	
Signet ring cells	296 (14.9)	151 (16.5)	145 (13.6)	
Other	21 (1.1)	9 (1.0)	12 (1.1)	0.18 ^a^
**Tumor location**				
Gastric cancer (non-cardia)	1365 (68.9)	677 (74.2)	688 (64.4)	
GEJ/cardia	558 (28.2)	219 (24.0)	339 (31.7)	
Distal esophagus	59 (3.0)	17 (1.9)	42 (3.9)	<0.001 ^a^
**Differentiation grade**				
Well differentiated (G1)	35 (1.8)	18 (2.0)	17 (1.6)	
Moderately differentiated (G2)	544 (27.4)	228 (25.0)	316 (29.6)	
Poorly differentiated (G3)	1029 (51.9)	476 (52.1)	553 (51.7)	
Undifferentiated (G4)	13 (0.7)	8 (0.9)	5 (0.5)	
Grade cannot be assessed (Gx)	361 (18.2)	183 (20.0)	178 (16.7)	0.07 ^a^
**Lauren classification**				
Intestinal	889 (44.9)	360 (39.4)	529 (49.5)	
Diffuse	744 (37.5)	363 (39.8)	381 (35.6)	
Mixed	85 (4.3)	35 (3.8)	50 (4.7)	
Unknown	264 (13.3)	155 (17.0)	109 (10.2)	<0.001 ^a^
**Clinical T-stage**				
cT2	573 (28.9)	330 (36.1)	243 (22.7)	
cT3	1052 (53.1)	390 (42.7)	662 (61.9)	
cT4a	131 (6.6)	46 (5.0)	85 (8.0)	
cTx	226 (11.4)	147 (16.1)	79 (7.4)	<0.001 ^a^
**Clinical N-stage**				
cN0	1009 (50.9)	480 (52.6)	529 (49.5)	
cN1	575 (29)	249 (27.3)	326 (30.5)	
cN2	308 (15.5)	146 (16.0)	162 (15.2)	
cN3	28 (1.4)	9 (1.0)	19 (1.8)	
cNx	62 (3.1)	29 (3.2)	33 (3.1)	0.17 ^a^
**WHO performance status**				
0	940 (47.4)	403 (44.1)	537 (50.2)	
1	667 (33.7)	293 (32.1)	374 (35.0)	
2–4	67 (3.4)	28 (3.1)	39 (3.6)	
Unknown	308 (15.5)	189 (20.7)	119 (11.1)	<0.001 ^a^
**Number of comorbidities**				
0	1032 (52.1)	477 (52.2)	555 (51.9)	
1–2	784 (39.6)	347 (38.0)	437 (40.9)	
>2	80 (4.0)	33 (3.6)	47 (4.4)	
Unknown	86 (4.3)	56 (6.1)	30 (2.8)	0.002 ^a^
**Diagnostic staging laparoscopy**	1196 (60.3)	403 (44.1)	793 (74.2)	<0.001 ^a^

EGJ: esophagogastric junction; IQR: interquartile range; FLOT: fluorouracil, leucovorin, oxaliplatin, and docetaxel; ^a^ Chi-square *p*-value; ^b^ Kruskal–Wallis *p*-value.

**Table 2:** Univariate and multivariate Cox regression analysis for survival after neoadjuvant anthracyclin triplets or FLOT chemotherapy.

	Univariate Analysis HR (95% CI)	*p-*Value	Multivariate Analysis HR (95% CI)	*p-*Value
**Sex**				
Male	1 [reference]			
Female	0.97 (0.85–1.11)	0.64		
**Age**	1.01 (1.01–1.02)	<0.0001	1.02 (1.01–1.03)	<0.0001
**Tumor type**				
Adenocarcinoma	1 [reference]			
Signet ring cells	1.08 (0.91–1.29)	0.36		
Other/unknown	1.73 (0.99–2.98)	0.05		
**Tumor location**				
Gastric cancer (non-cardia)	1 [reference]			
GEJ/cardia	1.01 (0.88–1.16)	0.93		
Distal esophagus	1.31 (0.92–1.87)	0.13		
**Differentiation grade**				
G1–G2	1 [reference]			
G3–G4	1.61 (1.38–1.88)	<0.0001	1.31 (1.09–1.56)	0.004
Unknown/missing	1.79 (1.48–2.17)	<0.0001	1.48 (1.20–1.83)	0.0002
**Lauren classification**				
Intestinal	1 [reference]			
Diffuse	1.72 (1.50–1.98)	<0.0001	1.73 (1.47–2.05)	<0.0001
Mixed	1.35 (0.99–1.85)	0.06	1.34 (0.97–1.84)	0.08
Unknown	1.35 (1.11–1.64)	0.003	1.19 (0.97–1.46)	0.09
**cT-category**				
cT2	1 [reference]			
cT3	1.23 (1.07–1.43)	0.005	1.27 (1.09–1.49)	0.002
cT4a	1.44 (1.11–1.87)	0.006	1.38 (1.06–1.80)	0.02
cTx	0.88 (0.70–1.10)	0.25	0.89 (0.71–1.11)	0.29
**cN-category**				
cN0	1 [reference]			
cN+	1.36 (1.19–1.54)	<0.0001	1.39 (1.22–1.59)	<0.0001
cNx	1.51 (1.08–2.09)	0.02	1.57 (1.12–2.19)	0.009
**WHO performance status**				
0–1	1 [reference]			
2–4	1.71 (1.27–2.30)	0.0004	1.70 (1.26–2.30)	0.0005
Unknown	1.12 (0.95–1.33)	0.16	1.12 (0.94–1.32)	0.2
**Number of comorbidities**				
0	1 [reference]			
1–2	0.94 (0.82–1.07)	0.36		
>2	1.22 (0.90–1.65)	0.21		
Unknown	1.12 (0.84–1.49)	0.45		
**Diagnostic laparoscopy**				
No	1 [reference]			
Yes	1.14 (1.00–1.30)	0.04	1.03 (0.90–1.19)	0.65

**Table 3:** Secondary outcomes for patients treated with anthracyclin triplets or FLOT chemotherapy.

	Anthracyclin Triplets (*n* = 797, %)	FLOT (*n* = 951, %)	Adjusted or FLOT (95% CI)	Multivariable Adjusted *p*-Value
Completed neoadjuvant chemotherapy	667 (73.1)	829 (77.6)	1.32 (1.05–1.66)	0.02
Resection after neoadjuvant treatment	797 (87.3)	951 (89.0)	1.34 (0.98–1.82)	0.07
Radical resection (R0)	698 (87.6)	832 (87.5)	1.24 (0.88–1.73)	0.22
Pathological complete response	62 (7.8)	93 (9.8)	1.58 (1.08–2.32)	0.02
ypT0-1	156 (19.6)	227 (23.9)	1.86 (1.43–2.42)	<0.0001
ypN0	344 (43.2)	448 (47.1)	1.50 (1.20–1.88)	0.0003
30-day mortality 90-day mortality	20 (2.5) 32 (4.0)	8 (0.8) 23 (2.4)	0.32 (0.12–0.81) 0.56 (0.30–1.04)	0.02 0.07
Adjuvant therapy	468 (58.9)	611 (64.3)	1.37 (1.10–1.71)	0.005

FLOT: fluorouracil, leucovorin, oxaliplatin, and docetaxel; odds ratio (OR) adjusted for age, sex, performance status, number of comorbidities, clinical T- and N-stage, tumor location, Lauren classification, differentiation grade, morphology, and undergoing diagnostic staging laparoscopy before treatment.

## Data Availability

The data underlying this article were provided by the Netherlands Comprehensive Cancer Organization (IKNL). Data can be shared according to regular procedures of the Netherlands Cancer Registry (NCR) upon reasonable request.

## Supplementary Materials

The following supporting information can be downloaded at: https://www.mdpi.com/article/10.3390/cancers16071291/s1, Table S1: Patient and tumor characteristics of the current study and the FLOT4-AIO trial; Table S2: Patient and tumor characteristics; Table S3: Number of patients who underwent primary resection over the past years.
