# Supplementary material for: Perioperative Chemotherapy for Gastro-Esophageal or Gastric Cancer: Anthracyclin Triplets versus FLOT
Source: Cancers (Basel). 2024 Mar 26;16(7):1291. doi: 10.3390/cancers16071291 (PMC11011191; doi:10.3390/cancers16071291)
Supplement: Supplementary file 1 [file cancers-16-01291-s001.zip › cancers-2912306-supplementary.pdf]

## Supplementary tables and figures

## Table S1

Patient and tumor characteristics of the current study and the FLOT4-AIO trial

| | This study | | FLOT4-AIO trial | |
|---|---|---|---|---|
| **Characteristics** | **Anthracyclin triplets (*n*= 913, %)** | **FLOT (*n*= 1069, %)** | **ECF/ECX (*n*= 360, %)** | **FLOT (*n*= 356, %)** |
| **Sex** | | | | |
| Male | 597 (65) | 733 (69) | 265 (74) | 268 (75) |
| **Age** (median [range]) | 66 (59 – 72) | 67 (59 – 73) | 62 (52 – 69) | 62 (54 – 69) |
| **Tumor type** | | | | |
| Adenocarcinoma | 753 (83) | 912 (85) | 259 (72) | 256 (72) |
| Signet ring cells | 151 (17) | 145 (14) | 101 (28) | 100 (28) |
| Other | 9 (1) | 12 (1) | - | - |
| **Lauren classification** | | | | |
| Diffuse | 363 (40) | 381 (36) | 96 (27) | 95 (27) |
| Intestinal or mixed | 395 (43) | 579 (54) | 163 (45) | 159 (45) |
| Unknown | 155 (17) | 109 (10) | 101 (28) | 102 (29) |
| **Clinical T-stage** | | | | |
| cT1 | - | - | 2 (1) | 3 (1) |
| cT2 | 330 (36) | 243 (23) | 59 (16) | 49 (14) |
| cT3 | 390 (43) | 662 (62) | 253 (70) | 267 (75) |
| cT4a | 46 (5) | 85 (8) | 33 (9) | 28 (8) |
| cTx | 147 (16) | 79 (7) | 13 (4) | 9 (3) |
| **Clinical N-stage** | | | | |
| cN0 | 480 (53) | 529 (50) | 70 (19) | 77 (22) |
| cN+ | 404 (44) | 507 (47) | 290 (81) | 279 (78) |
| Unknown | 29 (3) | 33 (3) | - | - |
| **WHO performance status** | | | | |
| 0 | 403 (44) | 537 (50) | 254 (71) | 246 (69) |
| 1 | 293 (32) | 374 (35) | 103 (29) | 109 (31) |
| 2 | 26 (3) | 30 (3) | 3 (1) | 1 (<1) |
| 3-4 | 2 (<1) | 9 (1) | - | |
| Unknown | 189 (21) | 119 (11) | | |

FLOT: fluorouracil, leucovorin, oxaliplatin and docetaxel; ECF: epirubicin/cisplatin/5-fluorouracil; ECX: epirubicin/cisplatin/capecitabin

**Table S2**

Patient and tumor characteristics

| Characteristics | Anthracyclin triplets (*n*= 913, %) | FLOT (*n*= 1069, %) | Primary resection (*n*= 1053, %) | *P-value* |
|---|---|---|---|---|
| **Sex** | | | | |
| Male | 597 (65.4) | 733 (68.6) | 656 (62.3) | ***0.010*** [a] |
| **Age** (median [IQR]) | 66 (59 – 72) | 67 (59 – 73) | 78 (71 – 82) | *N/A* |
| **Tumor type** | | | | |
| Adenocarcinoma | 753 (82.5) | 912 (85.3) | 872 (82.8) | |
| Signet ring cells | 151 (16.5) | 145 (13.6) | 144 (13.7) | |
| Other | 9 (1.0) | 12 (1.1) | 37 (3.5) | ***<0.001*** [a] |
| **Differentiation grade** | | | | |
| Well differentiated (G1) | 18 (2.0) | 17 (1.6) | 13 (1.2) | |
| Moderately differentiated | 228 (25.0) | 316 (29.6) | 386 (36.7) | |
| (G2) | 476 (52.1) | 553 (51.7) | 550 (52.2) | |
| Poorly differentiated (G3) | 8 (0.9) | 5 (0.5) | 11 (1.0) | |
| Undifferentiated (G4) | 183 (20.0) | 178 (16.7) | 93 (8.8) | ***<0.001*** [a] |
| Grade cannot be assessed (Gx) | | | | |
| **Lauren classification** | | | | |
| Intestinal | 360 (39.4) | 529 (49.5) | 573 (56.4) | |
| Diffuse | 363 (39.8) | 381 (35.6) | 293 (28.8) | |
| Mixed | 35 (3.8) | 50 (4.7) | 45 (4.4) | |
| Unknown | 155 (17.0) | 109 (10.2) | 142 (13.5) | ***<0.001*** [a] |
| **Clinical T-stage** | | | | |
| cT2 | 330 (36.1) | 243 (22.7) | 370 (35.1) | |
| cT3 | 390 (42.7) | 662 (61.9) | 367 (34.9) | |
| cT4a | 46 (5.0) | 85 (8.0) | 38 (3.6) | |
| cTx | 147 (16.1) | 79 (7.4) | 278 (26.4) | ***<0.001*** [a] |
| **Clinical N-stage** | | | | |
| cN0 | 480 (52.6) | 529 (49.5) | 736 (69.9) | |
| cN1 | 249 (27.3) | 326 (30.5) | 193 (18.3) | |
| cN2 | 146 (16.0) | 162 (15.2) | 77 (7.3) | |
| cN3 | 9 (1.0) | 19 (1.8) | 2 (0.2) | |
| cN3a | - | - | 1 (0.1) | |
| cNx | 29 (3.2) | 33 (3.1) | 44 (4.2) | ***<0.001*** [a] |
| **WHO performance status** | | | | |
| 0 | 403 (44.1) | 537 (50.2) | 246 (23.4) | |
| 1 | 293 (32.1) | 374 (35.0) | 272 (25.8) | |
| 2-4 | 28 (3.1) | 39 (3.6) | 147 (14.0) | |
| Unknown | 189 (20.7) | 119 (11.1) | 388 (36.8) | ***<0.001*** [a] |
| **Number of comorbidities** | | | | |
| 0 | 477 (52.2) | 555 (51.9) | 336 (31.9) | |
| 1 | 347 (38.0) | 437 (40.9) | 361 (34.3) | |
| ≥2 | 33 (3.6) | 47 (4.4) | 302 (28.7) | |

| | | | | |
|---|---|---|---|---|
| Unknown | 56 (6.1) | 30 (2.8) | 54 (5.1) | ***<0.001*** [a] |

N/A: not applicable;
[a] Chi-square p-value

**Table S3**

Number of patients who underwent primary resection over the past years

| Year of treatment | Number of patients who underwent primary resection | Number of patients who underwent neoadjuvant therapy (± resection) | Total number of patients | Percentage of primary resections |
|---|---|---|---|---|
| 2015 | 186 | 291 | 477 | 39.0% |
| 2016 | 176 | 280 | 456 | 38.6% |
| 2017 | 149 | 254 | 403 | 37.0% |
| 2018 | 146 | 310 | 456 | 32.0% |
| 2019 | 128 | 280 | 408 | 31.4% |
| 2020 | 148 | 276 | 424 | 34.9% |
| 2021 | 120 | 291 | 411 | 29.2% |
